# Emergency Ultrasound in the Context of Cardiac Arrest and Circulatory Shock: “How to Avoid Cardiac Arrest”

**DOI:** 10.3390/life15040646

**Published:** 2025-04-14

**Authors:** Rudolf Horn, Michael Blaivas, Daniel Wastl, Guido Michels, Armin Seibel, Susanne Morf, Marco Widler, Christoph F. Dietrich

**Affiliations:** 1 Center da sandà Val Müstair, 7536 Sta. Maria, Switzerland; rudolf.horn@csvm.ch (R.H.); susanne.morf@icloud.com (S.M.); 2 Department of Medicine, School of Medicine, University of South Carolina, Columbia, SC 29209, USA; mike@blaivas.org; 3 Bad Homburg Center of Intensive Care Medicine, Hochtaunus-Kliniken, 61352 Bad Homburg, Germany; daniel-wastl@t-online.de; 4 Notfallzentrum, Krankenhaus der Barmherzigen Brüder Trier, 54292 Trier, Germany; guidomichels@gmx.de; 5 Interdisciplinary Intensive Care Medicine, DRK Krankenhaus Kirchen, 57548 Kirchen, Germany; arminseibel1@me.com; 6 Department Allgemeine Innere Medizin (DAIM), Kliniken Hirslanden Beau Site, Salem und Permanence, 3013 Bern, Switzerland; f.dietrich@googlemail.com

**Keywords:** cardiopulmonary resuscitation, POCUS, hemodynamics, guideline, advanced cardiovascular life support (ACLS)

## Abstract

In the recently published 2021 European Resuscitation Council Guidelines on Adult Advanced Life Support, focused echocardiography was upgraded to a target recommendation. Several key recommendations were made, including that point-of-care ultrasound (POCUS) should only be used during CPR performed by experienced users and prolonged interruptions longer than 10 s (as accepted for pulse checking) during chest compressions should be avoided. Ultrasound does not replace clinical evaluation nor awareness of the clinical scenario. However, in addition to other assessments such as laboratory analyses, ultrasound can help to directly identify a cause for the peri-arrest state. The advantage of ultrasound is that examinations can be performed at the bedside while other tests are being carried out and repeated as frequently as required. This article focusses on how to use ultrasound during peri-arrest situations, requirements for ultrasound equipment, reversible causes of cardiac arrest, and the use of the RUSH protocol, focused echocardiography, and “deresuscitation” (post resuscitation/return of spontaneous circulation).

## 1. Introduction

In the recently published 2021 European Resuscitation Council Guidelines on Adult Advanced Life Support, focused echocardiography was upgraded to a target recommendation [1]. Several key recommendations were made, including that point-of-care ultrasound (POCUS) should only be used during CPR (cardiopulmonary resuscitation) performed by experienced users and prolonged interruptions longer than 10 s (as accepted for pulse checking) during chest compressions should be avoided.

There are three general uses of ultrasound in the peri-arrest situation. First, to look for a possible cause in a potential upcoming arrest situation so that cardiopulmonary arrest can be prevented. Second, to use during CPR to continue searching for the cause of arrest and to guide potential interventions, as well as to check cardiac activity during a compression pause. Third, to use in a phase of ROSC (return of spontaneous circulation) to guide therapy and find sources if the patient deteriorates.

Ultrasound gives us the unique opportunity to use an imaging-based examination during clinical evaluation to quickly identify potential causes of an impending cardiac arrest. In contrast to other imaging-based examinations, ultrasound examinations can be performed while the emergency patient continues to receive normal care. This makes ultrasound examination the only option for a rapidly performed bedside imaging [2,3]. Furthermore, responses to therapeutic interventions can be sought at will by repeating the ultrasound as needed [4]. One of the most tried and tested resuscitation-style algorithms is the RUSH protocol (rapid ultrasound for shock and hypotension) [4]. This review explores how the cause of an impending cardiac arrest can be recognized and, thus, treated before a cardiac arrest occurs. Furthermore, in the event of an ROSC, the cause must be sought again and addressed specifically so that circulation can be maintained. This paper deliberately does not focus on the underlying diseases that are encountered, but rather on a sonographically supported approach for patients in a peri-arrest situation presenting to the emergency department. Fortunately, there are now many studies that describe the benefits of sonography in peri-arrest situations [5,6,7,8,9].

## 2. Conditions That Can Lead to Cardiac Arrest

In cardiopulmonary resuscitation, including persistent ventricular fibrillation, the following reversible causes of cardiac arrest must be assessed (Table 1).

Fifty percent of these reversible causes can be identified on ultrasound imaging, potentially enabling immediate intervention. Sorted in order of significance, these are shown in Table 2.

In this list, which is based on the ACLS Guidelines (advanced cardiac life support), distributive shock is assigned to hypovolemia. Distributive shock is related primarily to sepsis or anaphylaxis. However, ultrasound does not provide any clear signs that prove distributive shock, but rather excludes other problems, which, together with the patient’s medical history and clinical findings, can result in a suspected diagnosis [5]. The five H’s and T’s, which can be detected by ultrasound, are also addressed in the RUSH protocol, which describes the sonographic procedure for evaluating the four classic groups of shock.

## 3. Requirements for Ultrasound Equipment

Most of an ultrasound examination can be performed with a single convex or sector transducer. However, when possible, the parallel use of convex, linear, and sector probes is ideal for different components of the examination. Each transducer type is ideal for certain portions of the evaluation, and the use of optimal presets can improve image quality and save time over manual setting adjustment. Another possibility is the use of a multidetector probe (foreseen with some handheld ultrasound devices), as it can be changed between the different applications. If there is only one probe, the sector probe is preferred (Table 3).

## 4. How to Use Ultrasound During Peri-Arrest Situation

Recently, many articles have appeared regarding innovations and clarifications in cardiopulmonary resuscitation. One question that still lingers is about hypothermia after cardiac arrest of 32–34 degrees over 24 h. In the most recent and comprehensive Cochrane systematic review and meta-analyses, including all RCTs, the beneficial effect of hypothermia in the range from 32 to 34 °C compared to normothermia or no temperature control was statistically significant [5]. Interestingly, significantly more ROSCs may occur during the spring and in the workplace than in other seasons or other locations [10,11,12,13]. A systematic review and meta-analysis showed that Amiodarone and lidocaine equally improved survival at hospital admission as compared with a placebo. However, neither amiodarone nor lidocaine improved long-term outcomes [14]. In addition to all the innovations in cardiopulmonary resuscitation, ultrasound is now available, which, thanks to its widespread availability, is ideally suited to identifying the causes of an impending cardiac arrest and initiating appropriate measures at an early stage.

Some physicians suggest that ultrasound in the peri-arrest situation interferes with the resuscitation process. The primary problem with ultrasound use, highlighted by its detractors, is the potential for lost chest compression time during resuscitation. Yet, ultrasound is an accepted and frequently used method of diagnosis among emergency physicians, especially in critical cardiopulmonary situations. While poor or unfavorable conditions resulting from ongoing resuscitation are often criticized as making ultrasound use difficult, ultrasound must be adapted to the diagnostic and therapeutic procedure in the peri-arrest situation and not vice versa. It is, therefore, necessary that the sonologist is able guide the probe with either their left or right hand, so that it is irrelevant on which side of the patient the machine stands, allowing it to be out of the way of resuscitative efforts. Ultrasound belongs to the attending physician, as does the stethoscope. A patient should never be brought to the ultrasound machine—the ultrasound machine has to be brought to the patient. When the ultrasound examination is performed by the attending physician, its findings can be directly linked to the patient’s clinical situation. Therefore, an ultrasound performed by a technician—as often practiced in organ sonography—is not equivalent. It is important to integrate ultrasound examination into the usual management process for an emergency patient. For example, the nurse decides which side has venous access. The team leader decides which sequence the examinations are to be carried out in and when the patient is ready for further examination, such as computed tomography. The ultrasound examination has a fixed place in the assessment, but the timing varies depending on the situation.

The treatment team should diagnose and treat the patient according to the ALS Guidelines (advanced life support). Ultrasound, positioned close to the patient, is used when a specific question needs to be answered. This process is well-known in trauma management with E-FAST (Extended Focused Assessment with Sonography for Trauma) examination. There is no other way to rapidly identify the presence of a large amount of free fluid in the thorax or abdomen, a pneumothorax, or pericardial effusion with a high sensitivity and specificity. Ultrasound should be used in much the same way in every peri-arrest situation without delay in the ALS process [2].

## 5. Examination Procedure

As always in critical situations, a clear working protocol helps to examine the patient quickly, without the omission of important parts. E.G. trauma patients are treated according to the ATLS principles, and a sonographic examination of an emergency patient should also be carried out according to clearly structured rules. Primarily, the examination should be divided into an infra- and supradiaphragmal examination. The supraphrenic examination includes a focused lung and heart ultrasound. There are also two subdiaphragmal types of examination, as follows: focused abdominal ultrasound and two-point sonography of the deep leg veins. Which examination is performed first depends on clinical probability. The organ that is likely to be the cause of the problem is examined first. The aim is to change the probe as little as possible in order to minimize the resulting loss of time.

## 6. RUSH—Protocol

The RUSH protocol [4] describes the four classic shock (hypovolemic, cardiogenic, obstructive, and distributive) states with their corresponding ultrasound findings. This protocol was able to correctly diagnose 100% of obstructive shock, 96.3% of cardiogenic shock, 94.4% of hypovolemic shock, 80.9% of the mixed type of shock, and 75% of the distributive type of shock [15].

The structures to be evaluated are classified as follows (Table 4):

- Pump: the heart (size and contractility of chambers), pericardial effusion, and cardiac thrombus.
- Tank: the inferior vena cava, jugular veins, peritoneal fluid, pleural fluid, lung with sliding, and lung rockets for pulmonary edema.
- Pipes: aortic aneurysm, aortic dissection, and deep vein thrombosis.

In a shocked patient, this protocol can be worked through in a few minutes. The start of the exam (first thorax, abdomen, or heart) depends on the clinical situation and the suspected disease. It is important that individual findings are not overestimated. The entire protocol should be worked through and as many findings as possible should be collected. Individual findings may be incidental (e.g., pre-existing abdominal aortic aneurysm) and should be interpreted in the clinical context.

## 7. Ultrasound Exam in Shock/Pre-Arrest

According to the RUSH protocol, the following sonographic examinations are required (Table 5):

### 7.1. LUS (Lung Ultrasound in Emergency Patients)

#### 7.1.1. Pneumothorax

One of the most important examinations is to rule in or out a pneumothorax. Ultrasound can be used to determine whether a patient has a pneumothorax and may reveal findings suggestive of a tension pneumothorax, such a cardiac shift or compression and a dilated IVC (inferior vena cava) with no respiratory variation. The patient’s vital signs and clinical scenario combine with ultrasound findings to arrive at a decision on the presence of a tension pneumothorax. The size of the pneumothorax can only be approximated by ultrasound, and this may add time to the examination.

The pneumothorax can be searched for using a convex, linear, or sector probe [16,17,18]. Post processing features (such as tissue harmonics, beam averaging, and others) should be turned off as much as possible. Failing to do so can make otherwise easily noted artifacts completely disappear. Further, a commonly seen mistake is forgetting to align a single focus zone with the pleural line, which can lead to artifact loss. Many modern point-of-care ultrasound machines have specific lung or pulmonary presets which optimize the imaging of lung artifacts [18,19].

**Figure 1:**
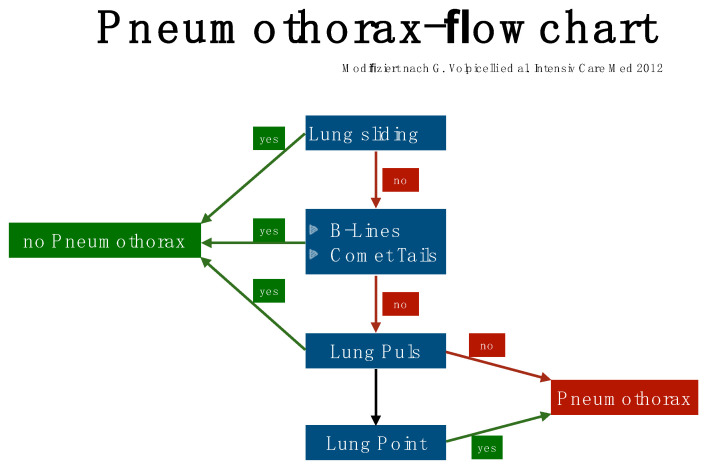
Pneumothorax flowchart modified according to Rudolf Horn [16].

**Figure 2:**
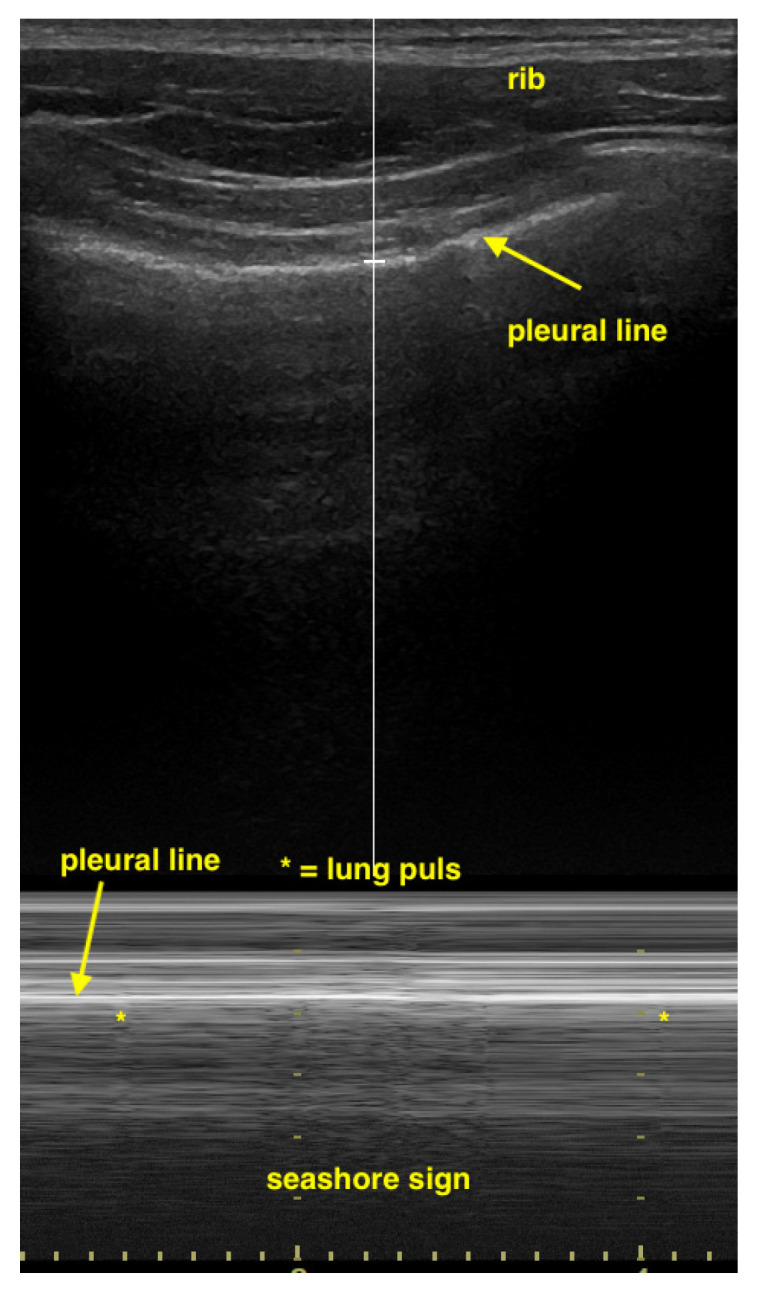
Sagittal view of pleural line behind the ribs. In the middle is the seashore sign, left and right of the lung pulse. The lung pulse in the middle cannot be differentiated from pleural movement which shows itself as seashore sign.

**Figure 3:**
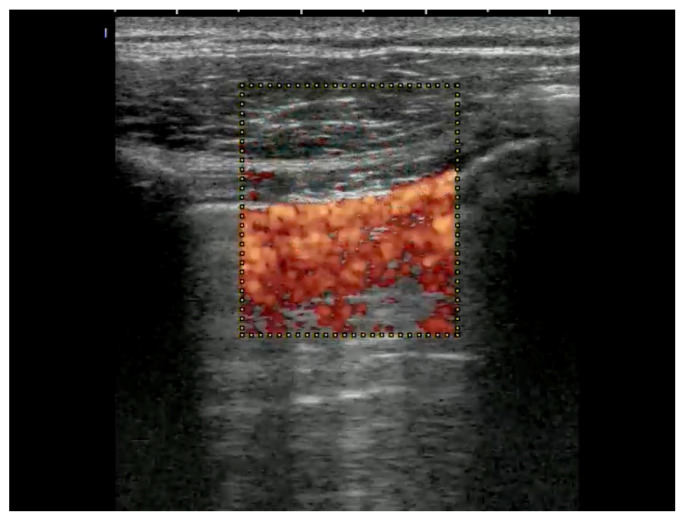
Color below pleural line means that there is movement because of breathing or lung pulse because of heart action.

Linear transducers will deliver the best depiction of the pleural line, with convex ones delivering a wider but less detailed view [19,20]. To detect a pneumothorax, the patient must be in a supine or half-sitting position (30–45 degrees). The position of the probe is parasagittal on both sides of the sternum on two rib interspaces close to the highest point of the thorax and on the anterior axillary line. Confirming lung sliding (sliding lung sign) is the first scanning task. The confirmation of visceral and parietal pleural movement in opposite directions is achieved by saving a cine loop, an image using M-Mode (seashore sign). If sliding cannot be definitively confirmed, the next step is to look for vertical artifacts and the lung pulse. Such vertical artifacts typically originate from the pleural line and will usually not be seen in the presence of a pneumothorax at that location. Lung pulse excludes the presence of pneumothorax at that location, but may indicate a lack of ventilation of the affected side. It can be visualized with M-Mode or color Doppler at and slightly below the pleural line (color sign). Of note, lung sliding, typically occurring in the top 2 to 4 cm of the image, is usually not seen as clearly with a sector transducer when compared with linear or large convex probes [21,22,23,24] (Table 5, Figure 1, Figure 2 and Figure 3).

#### 7.1.2. Pleural Effusion

**Figure 4:**
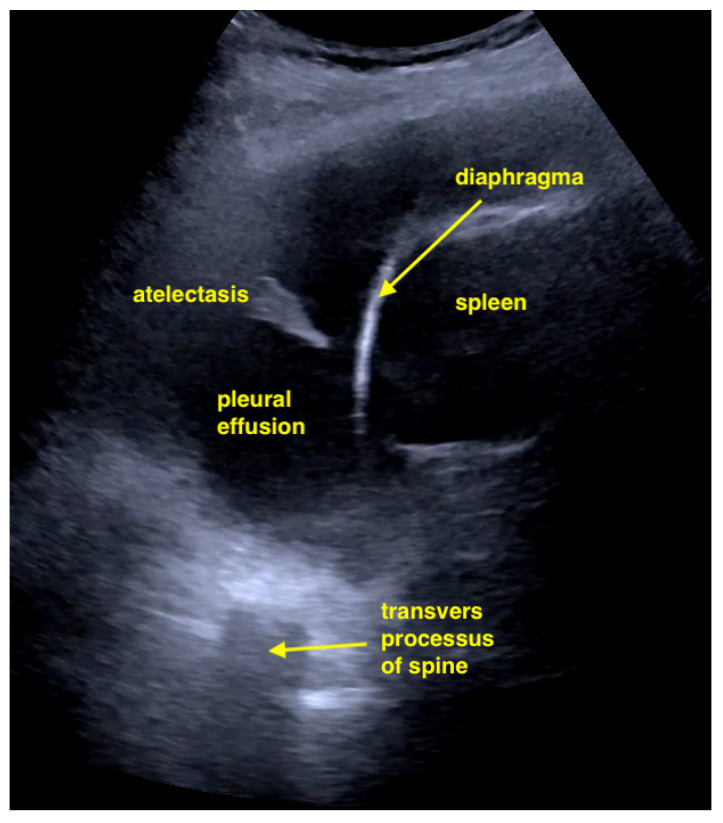
Large pleural effusion with compression atelectasis. If there is uncertainty as to whether it is a pleural effusion or not, the spine sign is shown. The transverse processes above the diaphragm are only visible if there is fluid in the pleural space. Otherwise, they are erased by lungs containing air.

Pleural effusion evaluation can be performed with either a sector or convex probe in the costophrenic angle on both sides of the thorax. Estimating the general size of an effusion (small versus large) is easy, however, obtaining a relatively precise volume estimate is difficult, with multiple different formulas available. Much more important is to realize whether an effusion is present [25] (Figure 4).

#### 7.1.3. Pulmonary Edema

**Figure 5:**
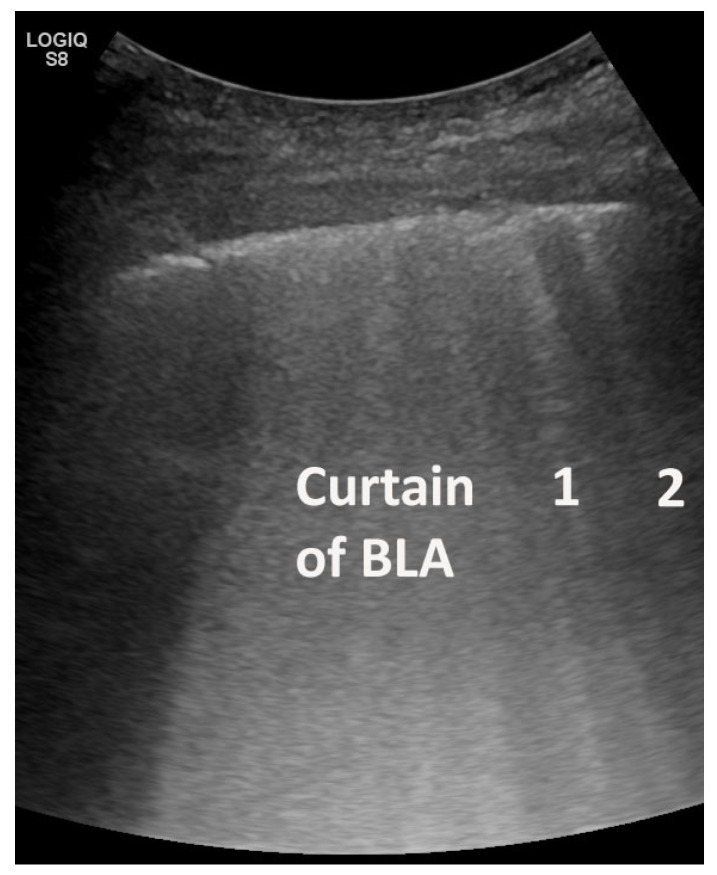
A cardiogenic pulmonary edema shows multiple B-line artifacts (BLAs) homogeneously distributed (as a “curtain” of BLA in the center of the image and lateral as individual BLA) bilaterally (evaluated by convex low frequency transducer <5 MHz without interfering presets). They arise from a smooth pleural line, are well defined, hyperechoic, extending indefinitely (at least 10 cm), and moving with lung sliding.

**Figure 6:**
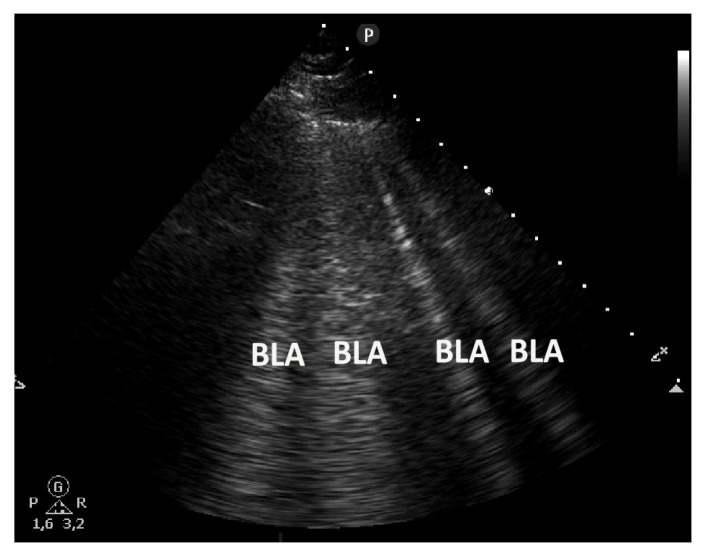
A cardiogenic pulmonary edema with multiple B lines (BLA) evaluated by sector transducer.

Pulmonary edema evaluation can be accomplished with a sector, convex, or even a linear transducer. Generally, evidence of edema is best seen with a sector probe, worst with a linear probe. Again, tissue harmonics, beam compounding, and any post processing should be turned off. Pulmonary edema manifests itself as a large amount (greater than two per rib interspace) of B-lines. In the supine patient, the thorax is divided into four areas on both sides. Ubiquitous interstitial syndrome has three or more B-lines in the sagittal view between two ribs, with this in two or more areas on both sides [18] (Figure 5 and Figure 6).

### 7.2. FOCUS (Focused Cardiac Ultrasound)

#### 7.2.1. Pericardial Effusion

**Figure 7:**
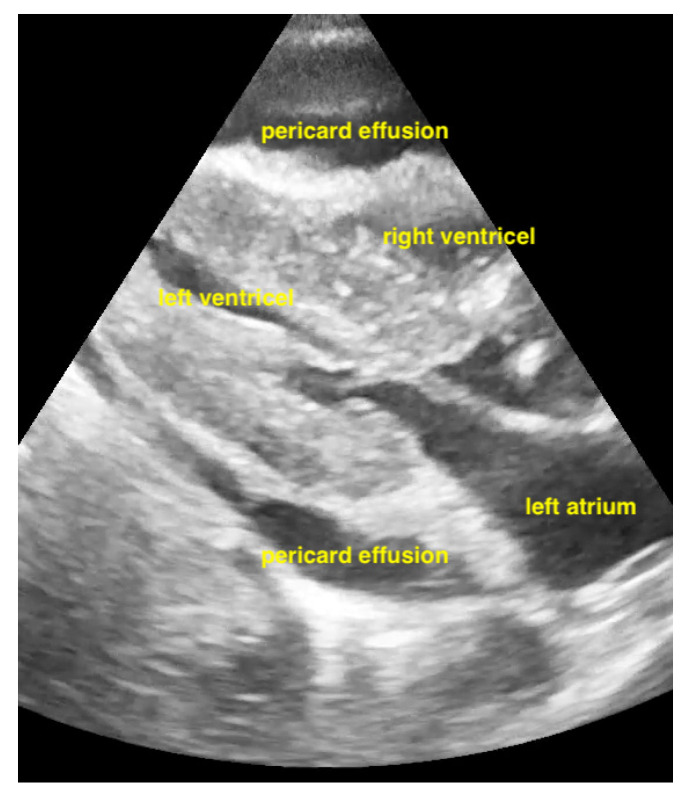
Pericardial effusion in parasternal long-axis view.

**Figure 8:**
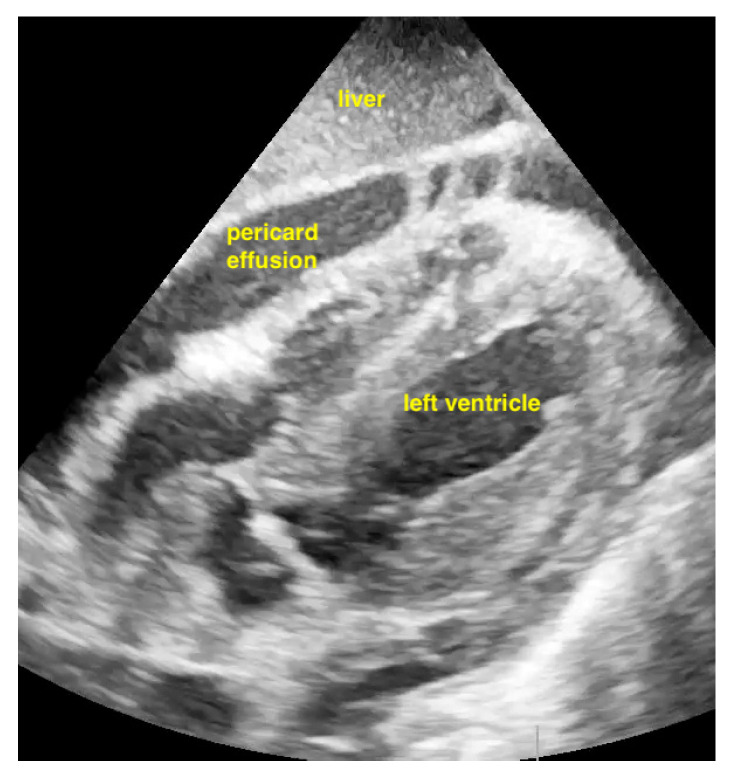
Pericardial effusion in subxiphoidal view.

**Figure 9:**
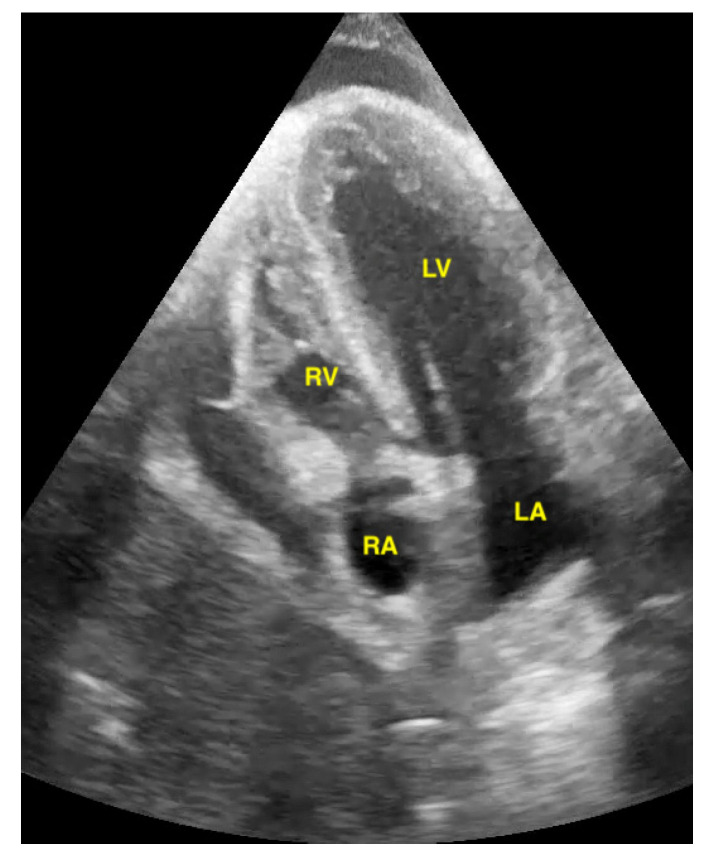
Pericardial effusion in apical four chamber view compresses the right atrium slightly (RA = right atrium, LA = left atrium, RV = right ventricle, LV = left ventricle).

**Figure 10:**
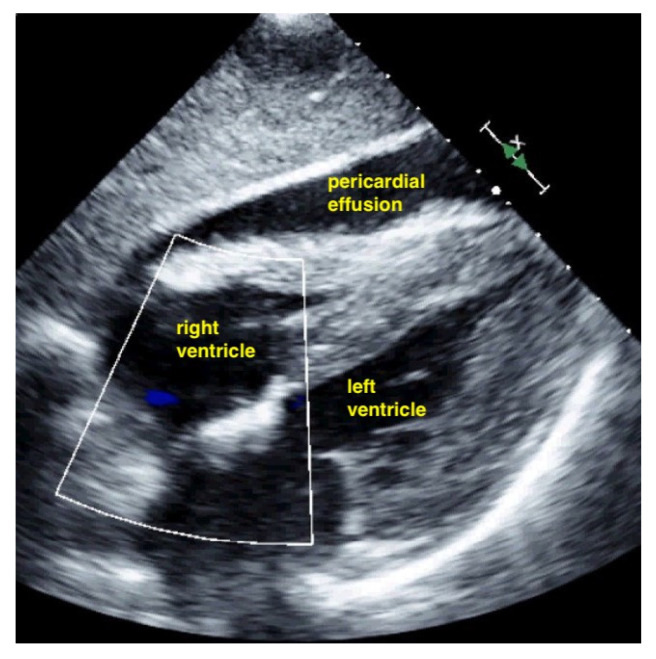
Pericardial effusion in subxiphoidal view compresses the right ventricle.

Pericardial effusions are best identified with a convex or sector probe in the subxiphoid or parasternal view. While it is important to simply note whether an effusion is present, sonographic and clinical (hypotension and tachycardia) evidence of cardiac tamponade should be sought. These include diastolic collapse of the right atrium free wall, followed by free wall collapse of the right ventricle as tamponade worsens. Both the apical and subcostal four-chamber views are ideal for this assessment. However, the subcostal window allows for a rapid transition to the proximal IVC view, which is dilated (>2 cm) and does not vary with respiration in cases of cardiac tamponade. Although many cases of medium and large pericardial effusions are obvious on ultrasound, there are notable exceptions. These include some hemorrhagic effusions which show significant coagulation, thus appearing echogenic on ultrasound, leading to frequent confusion with an epicardial fat pad or even a portion of the myocardial wall. Conversely, some epicardial fat pads may be confused with pericardial effusions, especially by novices. Differentiating between the two is easy by using anatomical knowledge. An epicardial fat pad does not extend enough inferiorly to be seen on a longitudinal IVC inlet view, while a pericardial effusion will in virtually all cases (Figure 7, Figure 8, Figure 9 and Figure 10).

**Figure 11:**
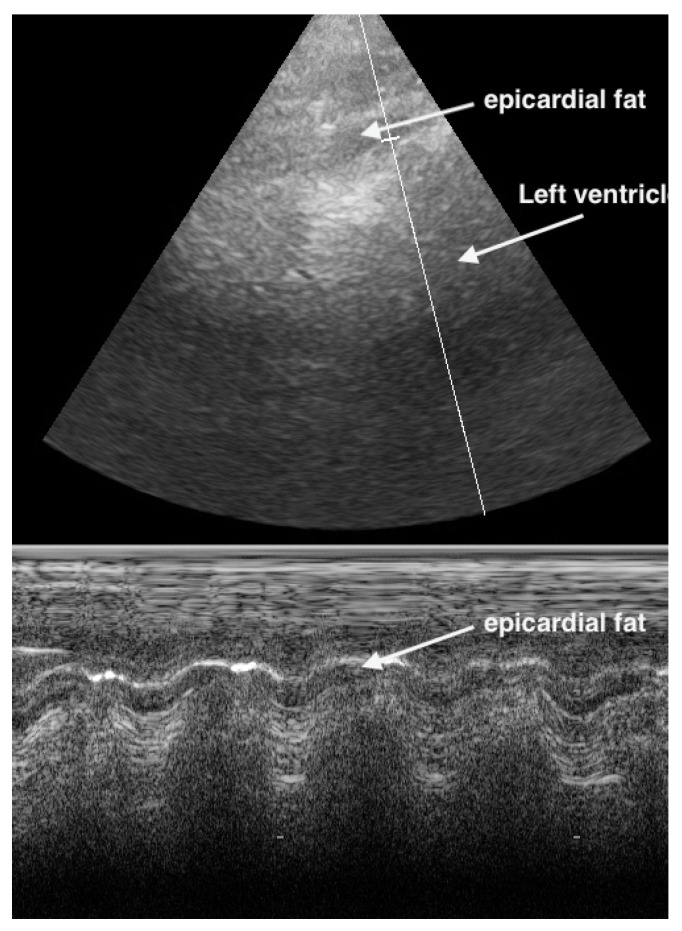
In the apical 4CV (Chambers almost not to see), in the apex is a broad hypoechoic band which has always the same size. 4CV = Four Chamber view.

**Figure 12:**
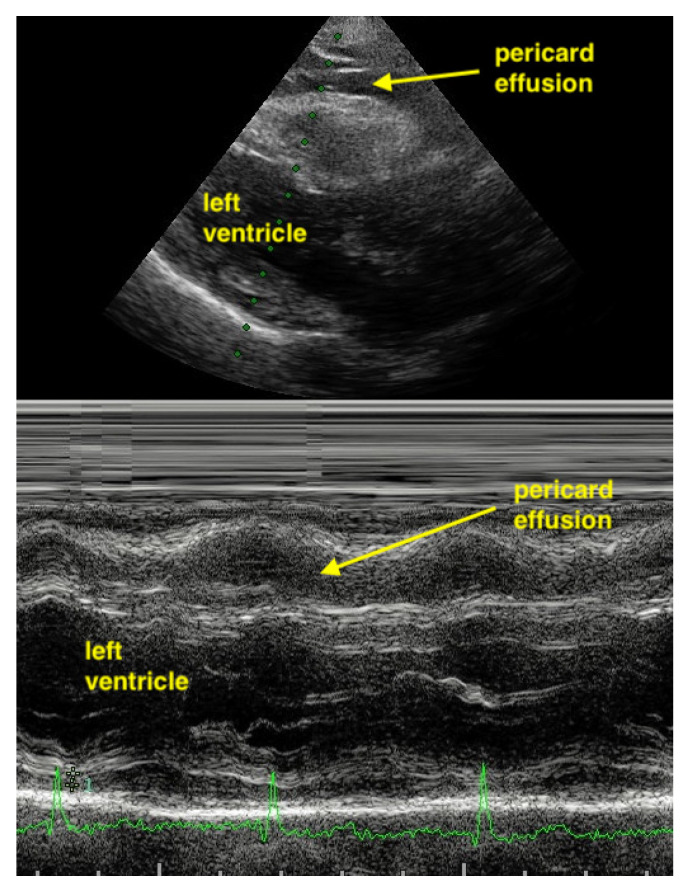
In the parasternal long-axis view, the hypoechoic band of the pericardial effusion varying in size during heart contraction (undulating).

M-Mode can help to differentiate between pericardial effusion and fat. Pericardial effusion undergoes a fluctuation during heart movement and so shows a hypoechoic band which moves in thickness. In contrast, the hypoechoic band of fat does not change in thickness (Figure 11 and Figure 12).

When cardiac tamponade from a pericardial effusion is suspected, immediate fluid drainage may be needed to avoid deterioration into full cardiac arrest. Traditionally, POCUS providers made use of a subcostal pericardiocentesis approach, but visualization and guidance of the needle tip are extremely challenging in this window.

A better approach is in the parasternal long axis, which allows for high-resolution imaging from the skin to the pericardial sac and myocardial wall. Ideally, pericardiocentesis in this location is performed using dynamic, in-plane needle guidance with a linear or higher-resolution convex transducer for the highest image resolution and real-time monitoring of the needle trajectory. Parasternal medial-to-lateral in-plane pericardiocentesis is also a new technique theoretically free of complications and enables the real-time monitoring of needle trajectory [26], but it is necessary to pay attention to the internal thoracic vessels (Table 6).

#### 7.2.2. Hypokinesis

**Figure 13:**
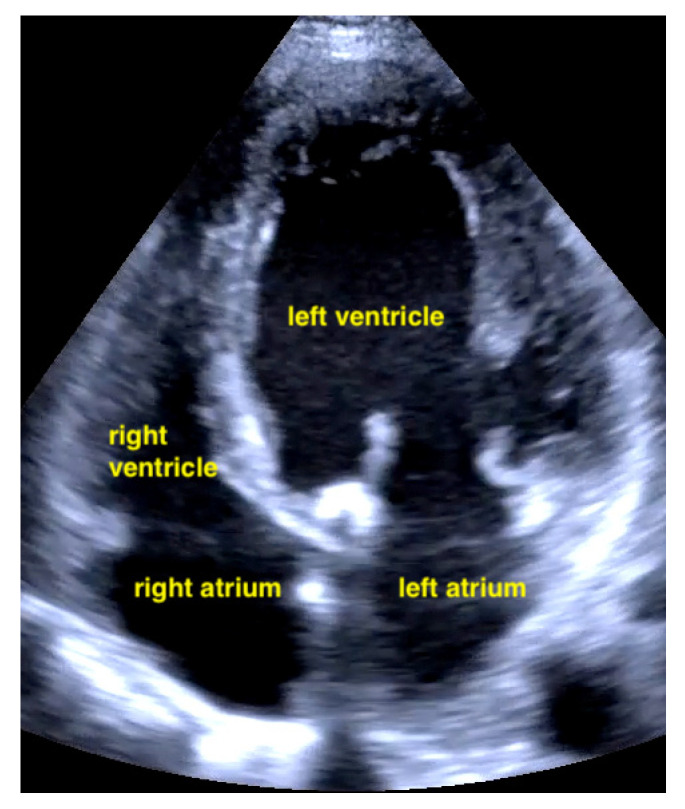
Enlarged left ventricle.

The left ventricle ejection fraction (LVEF) is determined using a sector probe in either the subcostal, parasternal, or apical views. In many critically ill patients, only one window may be available for EF assessment, and providers must make the best of a brief opportunity to obtain a qualitative determination of the EF. In this clinical situation, it is enough to appreciate the ejection fraction with eyeballing. With “eyeballing” (visual estimation), the following three grades are differentiated by novices: good (LVEF over 55%), restricted (LVEF 30–55%), and bad (LVEF < 30%) (Figure 13).

**Figure 14:**
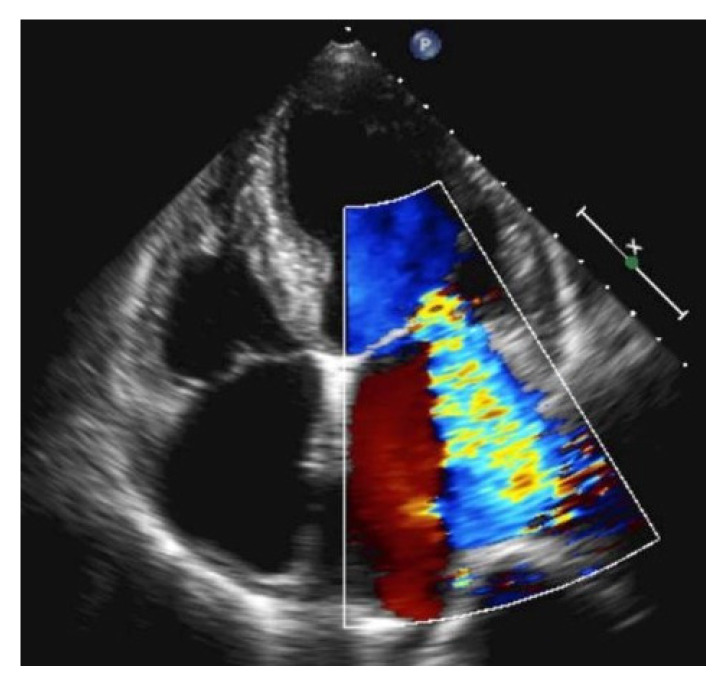
Large mitral regurgitation in apical four chamber view reaches the apex of the left atrium.

Recently, a number of POCUS devices have been including rapidly performed artificial intelligence (AI)-based EF assessment applications from one or more of the three windows. These AI EF applications can be highly accurate in real time when compared to comprehensive echocardiographic EF assessment and are likely to keep improving. Heart failure can also be caused by severe mitral regurgitation (e.g., papillary muscle rupture). In this case, the LVEF is normal or hyperdynamic. Severe mitral regurgitation can be seen with color Doppler over the mitral valve. In severe cases, the regurgitation jet reaches the apex of the left atrium (Figure 14).

Possible additional signs in left ventricular systolic dysfunction include the following:

- Pleural effusion.
- Pulmonary edema with ubiquitous B-Lines.
- Dilated left ventricle.
- Dilated atria.
- Inferior cava vein of >2 cm without respiratory diameter variance, often together with a secondary tricuspid regurgitation.
- All of these additional parameters alone.

#### 7.2.3. Pulmonary Embolism

**Figure 15:**
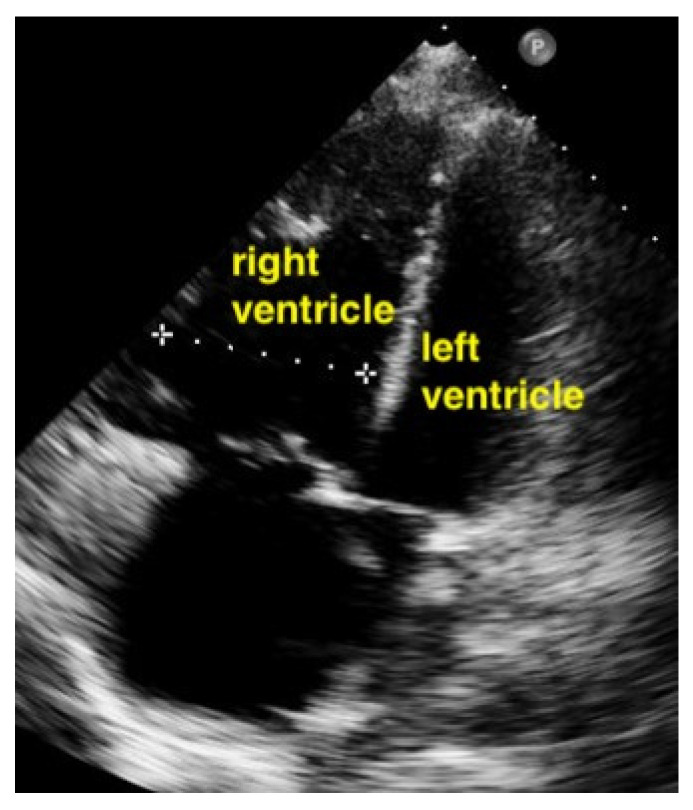
The right ventricle is much bigger than the left ventricle.

**Figure 16:**
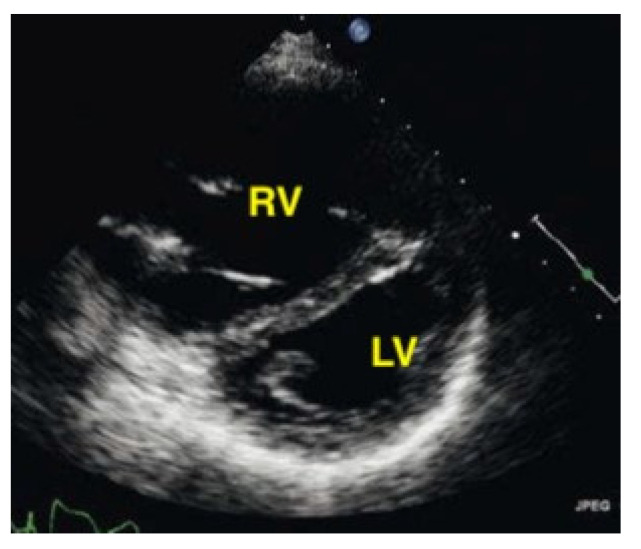
Parasternal short-axis view. The pathologically increased right ventricular pressure pushes the septum to the left side during diastole, so that a D-shape of the left ventricle results (RV = right ventricle, LV = left ventricle).

**Figure 17:**
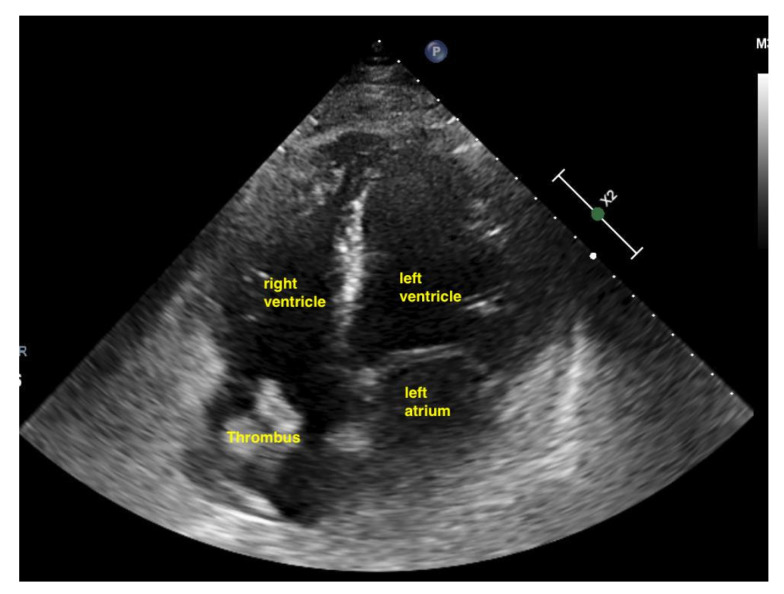
Thrombus in the right atrium in acute pulmonary embolism.

A pulmonary embolism large enough to compromise circulation is typically seen in more centrally located pulmonary arteries, and the subsequent pressure overload results in an enlarged right ventricle. Normally, the right ventricle is slightly smaller than the left ventricle. The size of the right ventricle compared to the left ventricle is best estimated in the apical four-chamber view with a sector probe. However, the subcostal four-chamber view will also reveal an unusually easily seen right ventricle when dilation is present and should be an indication to investigate further. In the case of a central pulmonary embolism, the interventricular septum is typically flattened (apical four-chamber view) and shows a D-sign (parasternal short-axis view) [29,30] (Figure 15, Figure 16 and Figure 17).

In this study, the authors evaluated the interobserver agreement of emergency physicians in detecting right ventricular dysfunction in pulmonary embolism. Criteria for right ventricular dysfunction in pulmonary embolism were defined as follows: (1) volume overload: the presence or absence of severe RV dilatation/enlargement (severe dilatation is defined as an RV/LV basal diameter ratio of 1.0 or higher and blunting of the apex of the RV from two or more different windows [McConnell sign]) and enlarged RV, often in combination with tricuspid regurgitation; (2) right heart myocardial disease: the presence or absence of severe RV systolic dysfunction (using tricuspid annular plane systolic excursion visual estimates of <10–(16) 18 mm and RV free-wall hypokinesis); and (3) pressure overload: the presence or absence of flattening or deviation of the interventricular septum toward the LV. The interobserver agreement for RV dysfunction by two emergency medicine residents was more 90%. Interobserver and Intraobserver Agreement on Qualitative Assessments of Right Ventricular Dysfunction with Echocardiography in Patients with Pulmonary Embolism [31].

Possible additional signs in pulmonary embolism include the following:

- Inferior cava vein of >2 cm without respiratory diameter variance.
- Deep vein thrombosis.
- Direct visualization of thrombus in the right heart cavities or pulmonary artery.

### 7.3. FAST (Focused Assessment with Sonography in Trauma)

#### Hypovolemia

In trauma, this examination is utilized to search for free fluid in the abdomen, presumed to be blood in most cases. However, this examination can be performed in general to look for free fluid in the abdomen, not just in trauma. The examination is performed using a convex or sector probe. The most important goal is to determine if free fluid is present in the abdomen. The dynamic change is important, what means repeating the examination, especially if the results are negative but the patient is still unstable or deteriorates. First, the upper right and left quadrants are examined. On the right side, fluid can be found in the perihepatic and subdiaphragmatic spaces, as well as in the Morison’s pouch. On the left side, fluid locations include the perisplenic and subdiaphragmatic spaces and the Koller pouch. In the pelvis, the peri vesicular spaces must be examined as well [32].

Possible additional signs in hypovolemia due to free fluid in abdomen include the following:

- Hyperdynamic left ventricle.
- Inferior cava vein of <1 cm and/or inspiratory collapse.
- Small size of cardiac cavities, sometimes seen as “kissing walls” of left ventricle.

### 7.4. Vessels

#### 7.4.1. Abdominal Aortic Aneurysm

**Figure 18:**
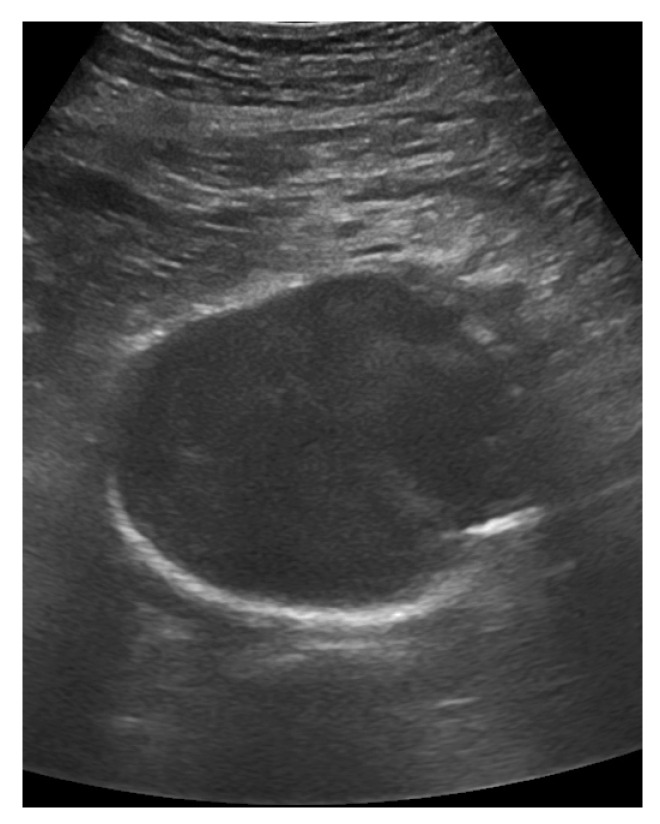
A large aortic aneurysm of 6.3 cm diameter.

An examination of the abdominal aorta can be performed with either a convex or sector probe [33]. The aorta is assessed for dilation with the ultrasound transducer in a transverse orientation. Traditionally, the diameter is measured in the anterior–posterior orientation, but saccular aneurysms should not be overlooked. A diameter of over 3 cm is considered to be an aortic aneurysm. A diameter of over 5 cm increases the risk for rupture (Figure 18). The most important risk factor for rupture is the diameter [34]. There is no measurement that proves a rupture, but for women with a diameter of more than 5 cm and for men with a diameter of more than 5,5 cm, the rupture risk increases, and, therefore, certain guidelines see this as an indication for surgery even if patients are asymptomatic. Any symptomatic aneurysm should be operated on [35]. Thus, in the event of shock and the presence of an aortic aneurysm without any other cause for shock, a ruptured aortic aneurysm must be considered. Hemorrhage is often retroperitoneal, rather than intraperitoneal, where it is difficult to identify. Possible additional signs in abdominal aortic aneurysm are summarized in the following sequence.

- Free fluid in abdomen.
- Enlarged retroperitoneum.
- Hyperdynamic left ventricle.
- Inferior cava vein of <1 cm.

#### 7.4.2. Aortic Dissection

A dissection of the aorta may not be associated with any dilation of the vessel, but it is potentially deadly nevertheless. While dissections may progress at varying rates, in an unstable patient found to have an aortic dissection, it is urgent to assess its extent. Dissection can affect low flow to vital organs such as the kidneys and the brain. One of the most deadly manifestations is a proximal aortic dissection coupled with a pericardial effusion (Type A dissection). The patient is at risk not only from tamponade due to the effusion, but the progression of the dissection to occlude coronary artery takeoff from the proximal aorta [36,37]. A dissection flap may be noted during an evaluation for a possible abdominal aortic aneurysm, this leads to the exploration of its extent.

Possible additional signs in aortic ascending dissection are as follows:

- Pericardial effusion/cardiac tamponade.
- Aortic regurgitation.
- Myocardial hypokinesis/left ventricular dysfunction suggestive of severe infarction.

#### 7.4.3. Deep Vein Thrombosis

**Figure 19:**
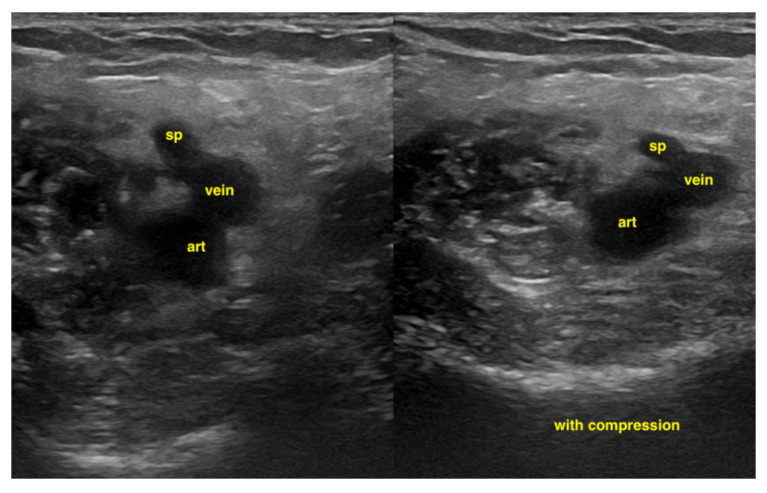
In the popliteal 4-region, the popliteal vein and the saphena parva vein cannot be compressed due to a thrombus.

An examination of the veins is usually performed with a linear transducer. Deep vein thrombosis is most reliably detected through compression sonography, although it may be obvious as an echogenic entity within the vessel lumen in some cases. The probe is in a transverse orientation with respect to the vessel. With pressure in a perpendicular vector on the vein, the vessel should collapse completely. It is important in this clinical situation to decide if there is a central pulmonary embolism too. In a peri-arrest situation, it is enough to apply a two-point compression ultrasound (inguinal and popliteal). Color Doppler is rarely required and would only be used to identify the location of vessels, as the examination relies solely on venous compression [38] (Figure 19). Possible additional signs in deep vein thrombosis can be found by looking for signs of central pulmonary embolism.

## 8. Limitation

However, ultrasound examinations have clear limitations. Not every patient presents good imaging windows. For example, in cachectic elderly patients, the heart may be very difficult to scan. Furthermore, patients in the emergency department are usually lying on their back. Sometimes, it is either impossible or takes a great deal of effort to turn the patient onto their left side for the FOCUS cardiac examination, potentially limiting imaging windows further. Likewise, the dorsal lung sections are best seen in a sitting position, which is rarely possible with emergency patients. The best equipment should be available in the emergency room, as the examination conditions are the worst here. However, it is often the case that the worst equipment is found in emergency rooms. Last but not least, ultrasound examinations are examiner-dependent. This applies to the examination technique, but also to the interpretation. It is important that, as with all instrumental examinations, ultrasound examinations are carried out according to the principle that the examination results must not be considered alone. The best results are achieved when the history, clinical picture, laboratory results, and ultrasound examination are considered together. Of course, it is absolutely necessary that this ultrasound examination must be learned well and practiced regularly. Without good control of the ultrasound probe, which must be practiced, results may disappoint [39].

## 9. “Deresuscitation” (Post Resuscitation/Return of Spontaneous Circulation)

After Return of Spontaneous Circulation (ROSC), frequently, a new chaos phase begins. The team has to check for or complete the search for reasons (e.g., 12 lead electrocardiogram). Simultaneously necessary medical interventions have to be completed if they have not been performed yet (secure airway; intravenous lines/intraosseous lines, complete monitoring; blood gas analysis, etc.). On the other hand, in many cases, patient transfer has to be prepared (from the scene to the hospital; the emergency room to the catheter lab; from the ward to intensive care unit; and so on). These aspects vary from patient to patient and situation to situation, requiring new organizations of the team with new individual assignments. Under no circumstances should sonography delay necessary (life-saving) measures. For example, coronary angiography should be performed immediately in the case of ST elevation myocardial infarction [40,41]. In the early phase after ROSC, sonography is a good way of finding or ruling out causes for resuscitation if this remains unclear (e.g., because there is no ST elevation or the medical history does not provide any good indications). For an experienced examiner, it makes sense to embed ultrasound in the above-mentioned assessment. From the authors’ point of view, the RUSH protocol is suitable for this, as it covers all the important compartments (lungs, heart, vessels, and abdomen), as described above. The examination should not take longer than 5 min and, if possible, should run in parallel with other necessary measures (authors’ opinion). The results are evaluated in a team time-out.

In the further course of care, the patient may become destabilized at any time. If causes remain unclear (e.g., problems with the airway or new malignant arrhythmia), the RUSH protocol can also help to clarify the situation. Particular attention should be paid to the consequences of resuscitation (pneumothorax, pericardial effusion, and liver or spleen injury).

Later on in the intensive care unit, sonography can be used to guide therapy. Sonography should be used during the daily ward round to focus on the volume status (respiratory variability of the vena cava and contraction behavior of the right ventricle (hyperdynamic) and the left ventricle (hyperdynamic), if it can do so). These results must not be considered in isolation, but must be placed in the context of the measured hemodynamic parameters, physical examination, and clinical course [42,43]. Echocardiography should at least be performed in a focused manner to record the course, for example, after myocardial infarction, but also to detect possible causes such as aortic valve stenosis or regional wall motion disorders in non-ST elevation myocardial infarction [44]. Focused echocardiography also takes time, so the patient must be stabilized and important therapeutic measures must have been completed. Transesophageal echocardiography is suitable for special questions (endocarditis) or difficult transthoracic conditions.

Even later on in the intensive care unit, the possibility of injury due to resuscitation must always be considered if deterioration occurs whose cause is not obvious (secondary spleen rupture). Sonography should also be the first diagnostic tool in this case [45,46].

## 10. Conclusions

The RUSH protocol, adapted to the sonographic examination procedure (LUS, FOCUS, FAST, Vessels), provides information about the cause of cardiopulmonary shock within a few minutes. Individual components of the examination may yield little information (e.g., dilated vena cava inferior without respiratory variability). However, taken as a whole, in the context of the state of shock and other parameters, this examination can provide information about the cause of shock. All sonographic findings, together with medical history and clinical findings, can result in a suspected diagnosis.

## Figures and Tables

**Table 1:** Reversible causes of cardiac arrest (5-H/5-T), according to the AHA-Algorithm.

Reversible Causes of Cardiac Arrest (5-H/5-T), According to the AHA-Algorithm	
Hypovolemia	Tension pneumothorax
Hypoxia	Tamponade (cardiac)
Hypothermia	Thrombosis (myocardial infarction)
H+ ions (acidosis)	Thrombosis (pulmonary)
Hypo-/hyperkalemia	Toxins

**Table 2:** Reversible causes of cardiac arrest revealed by ultrasound. Sorted in order of significance and treatability.

	Reversible Causes of Cardiac Arrest Revealed by Ultrasound. Sorted in Order of Significance and Treatability	Type of Shock
1	Tension pneumothorax	Obstructive shock
2	Tamponade (cardiac)	Obstructive shock
3	Hypovolemia	Hypovolemic/distributive shock
4	Thrombosis cardiac (myocardial infarction)	Cardiogenic shock
5	Thrombosis pulmonal (pulmonary embolism)	Obstructive/cardiogenic shock

**Table 3:** Settings.

Probe	Necessary Presets	Special Remarks
Abdominal convex	Abdomen	
	Lung	- Artifact suppression switched off - Color on PRF as low as possible
Linear probe	Small part (bone)	
	Lung	- Artifact suppression switched off - Color on PRF as low as possible
Sector probe	Echo Abdomen Lung	Handheld ultrasound devices are often sector scanners with broad frequencies which can be customized with various presets

**Table 4:** Rapid Ultrasound in Shock (RUSH) protocol—possible ultrasonographic findings seen with classic shock states.

RUSH Evaluation	Hypovolemic Shock	Cardiogenic Shock	Obstructive Shock	Distributive Shock
Pump	Hypercontractile heart Small chamber size	Hypo contractile heart Dilated heart	Hypercontractile heart Pericardial effusion Cardiac tamponade RV strain Cardiac thrombus	Hypercontractile heart (early sepsis) Hypo contractile heart (late sepsis)
Tank	Flat IVC Flat jugular veins Peritoneal fluid (fluid loss) Pleural fluid (fluid loss)	Distended IVC Distended jugular veins Lung rockets (pulmonary edema) Pleural fluid Peritoneal fluid (ascites)	Distended IVC Distended jugular veins Absent lung sliding (pneumothorax)	Normal or small IVC (early sepsis) Peritoneal fluid (sepsis source) Pleural fluid (sepsis source)
Pipes	Abdominal aneurysm Aortic dissection	Normal	DVT	Normal

Abbreviations: DVT = deep venous thrombosis, IVC = inferior vena cava, RV = right ventricle.

**Table 5:** Overview on the exam, probe, view, aim, and possible disease.

Exam	Probe	Possible Diseases	View	Look for
LUS	- Sector - Convex - Linear	Pneumothorax		- Lung sliding - B-Lines - Lung pulse
	- Sector - Convex	Pleural effusion	- Lateral - Dorsal	Costophrenic angle
	- Convex or Sector for B-Lines - Linear for pleura	Pulmonary edema	≥2 pos. regions on both sides	B-Lines, coming from intact pleura
FOCUS	Sector	Pericardial tamponade	- Subxiphoidal - Parasternal long-axis and short-axis	Pericard effusion
		Central pulmonary embolism	- Apical - Parasternal short-axis	- Size RV:LV - Septum flattening - D-Shape
		Hypokinesis	- Subxiphoid - Apical 4CV	LVEF
		Volume status	- VCI diameter and respiratory. Variability: subxiphoid	Volume status
FAST	- Sector - Convex	Free fluid in abdomen	- Right and left upper quadrant - lower abdomen/perivesical	Free fluid
Vessels	- Sector - Convex	Abdominal aortic aneurysm	Transverse view of aorta	
	Linear	Deep vein thrombosis	Inguinal and popliteal transverse view	Compression sonography

Abbreviations: LUS = lung ultrasound, RV = right ventricle, LV = left ventricle, FAST = Focused Assessment with Sonography for Trauma, 4 CV = four-chamber view, LVEF = left ventricular ejection fraction.

**Table 6:** Advantages and disadvantages of different pericardiocentesis approaches (modified from [27,28]).

Pericardiocentesis Approach	Advantages	Disadvantages
Subxiphoid	Extra pleural route	Liver injury Colon or stomach perforation Long distance to reach the pericardial cavity, especially in case of obesity Irritation to diaphragm or phrenic nerve
Left parasternal	Direct route to reach the pericardial cavity	Internal thoracic vessels injury Pneumothorax
Apical	Direct route to reach the pericardial cavity	Pneumothorax Ventricular apex piercing (ventricular arrhythmias)

## Data Availability

The original contributions presented in this study are included in the article. Further inquiries can be directed to the corresponding author.

## Abbreviations

FOCUS	Focused cardiac ultrasound
FAST	Focused assessment with sonography in trauma (can also be used without trauma)
IVC	Inferior vena cava
LV	Left ventricle
LUS	Lung ultrasound
LVEF	Left ventricle ejection fraction
PRF	Pulse Repetition Frequency
RV	Right ventricle

## References

[1] Soar J., Bottiger B.W., Carli P., Couper K., Deakin C.D., Djarv T., Lott C., Olasveengen T., Paal P., Pellis T. (2021). European Resuscitation Council Guidelines 2021: Adult advanced life support. Resuscitation.

[2] Goudie A., Blaivas M., Horn R., Lien W.C., Michels G., Wastl D., Dietrich C.F. (2024). Ultrasound during Advanced Life Support-Help or Harm?. Diagnostics.

[3] Wastl D., Blaivas M., Horn R., Michels G., Seibel A., Schwarz S., Hoffmann B.H., Hoffmann O., Von Ow D., Dietrich C.F. (2024). How to perform Point of Care Ultrasound at resuscitation and when it is useful. Med. Ultrason..

[4] Perera P., Mailhot T., Riley D., Mandavia D. (2010). The RUSH exam: Rapid ultrasound in shock in the evaluation of the critically lll. Emerg. Med. Clin. N. Am..

[5] Chua M.T., Chan G.W., Kuan W.S. (2017). Reversible Causes in Cardiovascular Collapse at the Emergency Department Using Ultrasonography (REVIVE-US). Ann. Acad. Med. Singap..

[6] Zollner K., Sellmann T., Wetzchewald D., Schwager H., Cleff C., Thal S.C., Marsch S. (2021). U SO CARE-The Impact of Cardiac Ultrasound during Cardiopulmonary Resuscitation: A Prospective Randomized Simulator-Based Trial. J. Clin. Med..

[7] Breitkreutz R., Price S., Steiger H.V., Seeger F.H., Ilper H., Ackermann H., Rudolph M., Uddin S., Weigand M.A., Muller E. (2010). Focused echocardiographic evaluation in life support and peri-resuscitation of emergency patients: A prospective trial. Resuscitation.

[8] Hafner C., Manschein V., Klaus D.A., Schaubmayr W., Tiboldi A., Scharner V., Gleiss A., Thal B., Krammel M., Hamp T. (2024). Live stream of prehospital point-of-care ultrasound during cardiopulmonary resuscitation—A feasibility trial. Resuscitation.

[9] Balderston J.R., You A.X., Evans D.P., Taylor L.A., Gertz Z.M. (2021). Feasibility of focused cardiac ultrasound during cardiac arrest in the emergency department. Cardiovasc. Ultrasound.

[10] Stirparo G., Andreassi A., Sechi G.M., Signorelli C. (2023). Spring, it’s time to ROSC. J. Prev. Med. Hyg..

[11] Hussein L., Rehman M.A., Sajid R., Annajjar F., Al-Janabi T. (2019). Bedside ultrasound in cardiac standstill: A clinical review. Ultrasound J..

[12] Blanco P., Martinez Buendia C. (2017). Point-of-care ultrasound in cardiopulmonary resuscitation: A concise review. J. Ultrasound.

[13] Avila-Reyes D., Acevedo-Cardona A.O., Gomez-Gonzalez J.F., Echeverry-Piedrahita D.R., Aguirre-Florez M., Giraldo-Diaconeasa A. (2021). Point-of-care ultrasound in cardiorespiratory arrest (POCUS-CA): Narrative review article. Ultrasound J..

[14] Sanfilippo F., Corredor C., Santonocito C., Panarello G., Arcadipane A., Ristagno G., Pellis T. (2016). Amiodarone or lidocaine for cardiac arrest: A systematic review and meta-analysis. Resuscitation.

[15] Bolder P.M., Norton M.L. (1984). Retinal hemorrhage following anesthesia. Anesthesiology.

[16] Horn R., Gorg C., Prosch H., Safai Zadeh E., Jenssen C., Dietrich C.F. (2024). Sonography of the pleura. Ultraschall Med..

[17] Dietrich C.F., Gorg C., Horn R., Prosch H., Safai Zadeh E., Jenssen C. (2023). Ultrasound of the lung. Ultraschall Med..

[18] Mathis G., Horn R., Morf S., Prosch H., Rovida S., Soldati G., Hoffmann B., Blaivas M., Dietrich C.F. (2021). WFUMB position paper on reverberation artefacts in lung ultrasound: B-lines or comet-tails?. Med. Ultrason..

[19] Dietrich C.F., Mathis G., Blaivas M., Volpicelli G., Seibel A., Wastl D., Atkinson N.S., Cui X.W., Fan M., Yi D. (2016). Lung B-line artefacts and their use. J. Thorac. Dis..

[20] Dietrich C.F., Mathis G., Blaivas M., Volpicelli G., Seibel A., Atkinson N.S., Cui X.W., Mei F., Schreiber-Dietrich D., Yi D. (2016). Lung artefacts and their use. Med. Ultrason..

[21] Demi L., Wolfram F., Klersy C., De Silvestri A., Ferretti V.V., Muller M., Miller D., Feletti F., Welnicki M., Buda N. (2023). New International Guidelines and Consensus on the Use of Lung Ultrasound. J. Ultrasound Med..

[22] Ketelaars R., Gulpinar E., Roes T., Kuut M., van Geffen G.J. (2018). Which ultrasound transducer type is best for diagnosing pneumothorax?. Crit. Ultrasound J..

[23] Wilkerson R.G., Stone M.B. (2010). Sensitivity of bedside ultrasound and supine anteroposterior chest radiographs for the identification of pneumothorax after blunt trauma. Acad. Emerg. Med..

[24] Schnell J., Beer M., Eggeling S., Gesierich W., Gottlieb J., Herth F.J.F., Hofmann H.S., Jany B., Kreuter M., Ley-Zaporozhan J. (2019). Management of Spontaneous Pneumothorax and Post-Interventional Pneumothorax: German S3 Guideline. Respiration.

[25] Dickman E., Terentiev V., Likourezos A., Derman A., Haines L. (2015). Extension of the Thoracic Spine Sign: A New Sonographic Marker of Pleural Effusion. J. Ultrasound Med..

[26] Osman A., Wan Chuan T., Ab Rahman J., Via G., Tavazzi G. (2018). Ultrasound-guided pericardiocentesis: A novel parasternal approach. Eur. J. Emerg. Med..

[27] Blanco P., Figueroa L., Menendez M.F., Berrueta B. (2022). Pericardiocentesis: Ultrasound guidance is essential. Ultrasound J..

[28] Loukas M., Walters A., Boon J.M., Welch T.P., Meiring J.H., Abrahams P.H. (2012). Pericardiocentesis: A clinical anatomy review. Clin. Anat..

[29] Alerhand S., Sundaram T., Gottlieb M. (2021). What are the echocardiographic findings of acute right ventricular strain that suggest pulmonary embolism?. Anaesth. Crit. Care Pain. Med..

[30] Yılmaz F., Tekin Y., Toprak N., Eyinç M.B., Arslan E.D. (2022). A case of massive pulmonary embolism causing cardiac arrest managed with successful systemic thrombolysis in the emergency department. Emer. Care J..

[31] Weekes A.J., Oh L., Thacker G., Johnson A.K., Runyon M., Rose G., Johnson T., Templin M., Norton H.J. (2016). Interobserver and Intraobserver Agreement on Qualitative Assessments of Right Ventricular Dysfunction with Echocardiography in Patients with Pulmonary Embolism. J. Ultrasound Med..

[32] Simanowski J.H. (2019). Blunt trauma and acute diseases of the abdomen and chest: Free fluid—What now?. Ultraschall Med..

[33] Czerny M., Grabenwoger M., Berger T., Aboyans V., Della Corte A., Chen E.P., Desai N.D., Dumfarth J., Elefteriades J.A., Etz C.D. (2024). EACTS/STS Guidelines for diagnosing and treating acute and chronic syndromes of the aortic organ. Eur. J. Cardiothorac. Surg..

[34] Anagnostakos J., Lal B.K. (2021). Abdominal aortic aneurysms. Prog. Cardiovasc. Dis..

[35] Chaikof E.L., Dalman R.L., Eskandari M.K., Jackson B.M., Lee W.A., Mansour M.A., Mastracci T.M., Mell M., Murad M.H., Nguyen L.L. (2018). The Society for Vascular Surgery practice guidelines on the care of patients with an abdominal aortic aneurysm. J. Vasc. Surg..

[36] Benson R.A., Meecham L., Fisher O., Loftus I.M. (2018). Ultrasound screening for abdominal aortic aneurysm: Current practice, challenges and controversies. Br. J. Radiol..

[37] Arnold M.J., Jonas C.E., Carter R.E. (2020). Point-of-Care Ultrasonography. Am. Fam. Physician.

[38] Zuker-Herman R., Ayalon Dangur I., Berant R., Sitt E.C., Baskin L., Shaya Y., Shiber S. (2018). Comparison between two-point and three-point compression ultrasound for the diagnosis of deep vein thrombosis. J. Thromb. Thrombolysis.

[39] Michels G., Zinke H., Mockel M., Hempel D., Busche C., Janssens U., Kluge S., Riessen R., Buerke M., Kelm M. (2017). Recommendations for education in ultrasound in medical intensive care and emergency medicine: Position paper of DGIIN, DEGUM and DGK. Med. Klin. Intensivmed. Notfmed.

[40] Via G., Hussain A., Wells M., Reardon R., ElBarbary M., Noble V.E., Tsung J.W., Neskovic A.N., Price S., Oren-Grinberg A. (2014). International evidence-based recommendations for focused cardiac ultrasound. J. Am. Soc. Echocardiogr..

[41] Werdan K., Buerke M., Geppert A., Thiele H., Zwissler B., Russ M., on behalf of the guideline group (2021). Infarction-Related Cardiogenic Shock- Diagnosis, Monitoring and Therapy-A German-Austrian S3 Guideline. Dtsch. Arztebl. Int..

[42] Habicher M., Zajonz T., Heringlake M., Boning A., Treskatsch S., Schirmer U., Markewitz A., Sander M. (2018). S3 guidelines on intensive medical care of cardiac surgery patients: Hemodynamic monitoring and cardiovascular system-an update. Anaesthesist.

[43] Janssens U. (2016). Hemodynamic monitoring of critically ill patients: Bedside integration of data. Med. Klin. Intensivmed. Notfmed.

[44] Michels G., Pfister R., Hempel D. (2018). Focused echocardiography in acute medicine. Med. Klin. Intensivmed. Notfmed.

[45] Malbrain M., Martin G., Ostermann M. (2022). Everything you need to know about deresuscitation. Intensive Care Med..

[46] Tullo G., Candelli M., Gasparrini I., Micci S., Franceschi F. (2023). Ultrasound in Sepsis and Septic Shock-From Diagnosis to Treatment. J. Clin. Med..

